# Simultaneous presentation of giant aneurysms of the coronary sinus and superior vena cava

**DOI:** 10.5830/CVJA-2016-031

**Published:** 2016

**Authors:** Cheng Yan, Gao Huanhuan, Zheng Zhelan, Mou Yun

**Affiliations:** Giant pericardial cyst: case report.echocardiography and vascular ultrasound Centre, the First affiliated hospital, College of medicine, Zhejiang university, hangzhou, China; Giant pericardial cyst: case report.echocardiography and vascular ultrasound Centre, the First affiliated hospital, College of medicine, Zhejiang university, hangzhou, China; Giant pericardial cyst: case report.echocardiography and vascular ultrasound Centre, the First affiliated hospital, College of medicine, Zhejiang university, hangzhou, Chin; Giant pericardial cyst: case report.echocardiography and vascular ultrasound Centre, the First affiliated hospital, College of medicine, Zhejiang university, hangzhou, China

**Keywords:** aneurysm, coronary sinus, superior vena cava, right heart failure

## Abstract

Aneurysms of the coronary sinus and superior vena cava are rare and their aetiologies remain controversial. Some studies have shown that these acquired venous aneurysms are caused by an increase in right atrial pressure, which may be related to right heart failure. However, few reports have provided direct evidence to support this hypothesis. We present a rare case of combined giant aneurysms of the coronary sinus and vena cava, diagnosed using multiple imaging modalities. This case strongly supports the hypothesis that right heart diastolic failure may be an important mechanism underlying the pathogenesis of combined giant aneurysms.

## Abstract

Venous aneurysms are rare and the simultaneous presentation of aneurysms localised in the coronary sinus (CS) and superior vena cava (SVC) has not been previously reported. Venous aneurysms may be congenital or secondary to anomalous drainage.^1^,^2^

Some studies have shown that acquired venous aneurysms are most likely caused by an increase in right atrial pressure and right heart failure.^3^ However few reports have provided direct evidence supporting this pathogenic mechanism.

Here, we present a case of giant aneurysms localised in both the CS and SVC. Our case provides strong evidence for the possible role of longstanding right heart failure during the pathogenesis of combined giant venous aneurysms.

## Case report

A 22-year-old woman was referred to our hospital because of a large mass in the left thorax, detected on chest X-ray. She had a long history of constrictive pericarditis and had undergone pericardiectomy seven years earlier.

On presentation, a physical examination revealed severe facial and lower-extremity oedema, hepatomegaly and ascites. She had no cardiac murmur and no cyanosis, with an oxygen saturation of 96.6% on room air.

**Fig. 1:**
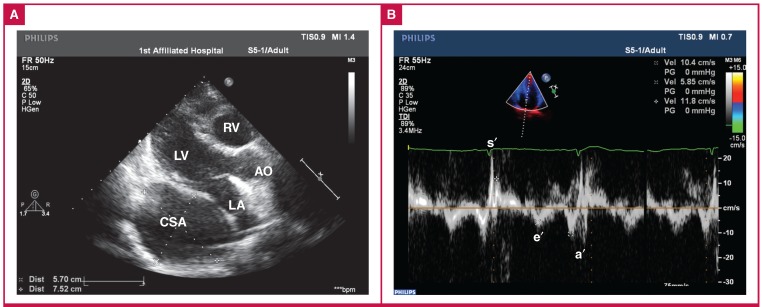
Transthoracic echocardiography on presentation showed: A. the appearance of the giant aneurysm of the coronary sinus in the parasternal long-axis view; B. tissue Doppler imaging velocities at the septal mitral annulus: early diastolic myocardial velocity (e′) was 5.85 cm/s and systolic myocardial velocity (s′) was 10.4 cm/s. RV, right ventricle; LA, left atrium; LV, left ventricle; CSA, coronary sinus aneurysm; AO, ascending artoa, a′, late diastolic myocardial velocity.

Transthoracic echocardiography showed an enlarged right atrium and a huge cavity behind the left heart (Fig. 1A). Pulsedwave Doppler identified a restrictive transmitral inflow pattern. Tissue Doppler imaging (TDI) velocities at the septal mitral annulus showed that early diastolic myocardial velocity (e′) and systolic myocardial velocity (s′) were 5.85 and 10.4 cm/s, respectively (Fig. 1B).

**Fig. 2:**
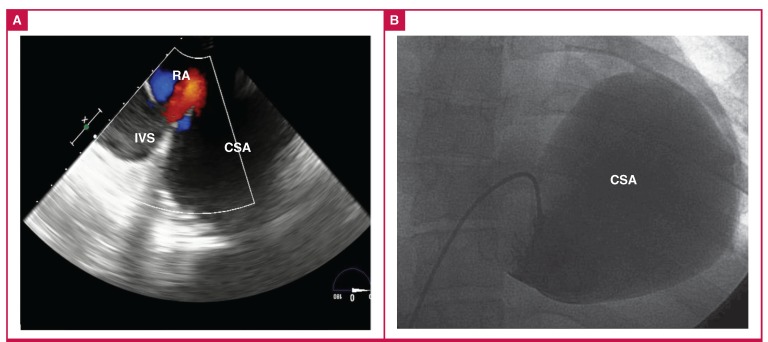
A. Transoesophageal echocardiography colour Doppler flow imaging demonstrated flow from the coronary sinus aneurysm to the right atrium. B. Cardiac catheterisation showed the appearance of the giant coronary sinus aneurysm. RA, right atrium; IVC, inferior vena cava; CSA, coronary sinus aneurysm.

**Fig. 3:**
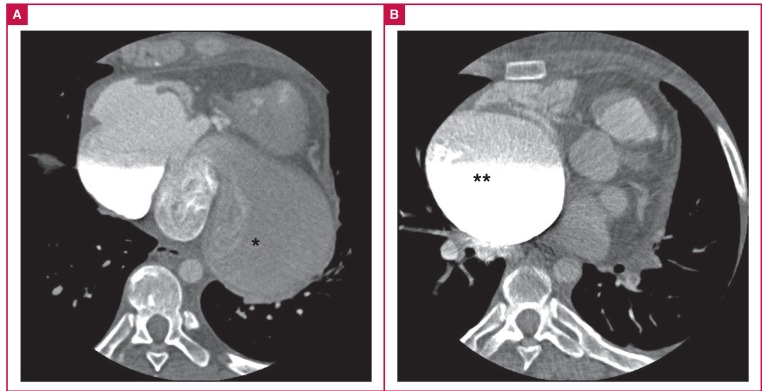
Contrast-enhanced CT showed a giant aneurysm of the coronary sinus (*) and a giant aneurysm of the superior vena cava (**).

Transoesophageal echocardiography revealed a large cystic cavity with spontaneous echo contrast attached to the posterior wall of the left heart and communication with the right atrium. Colour Doppler flow imaging demonstrated a to-and-fro flow between the cavity and right atrium (Fig. 2A). Contrastenhanced computed tomography (CT) showed a giant cavity (10.4 × 7.3 cm) connected with three cardiac veins (Fig. 3A) and revealed that it was an aneurysm of the CS. Moreover, CT showed no evidence of pericardial calcification or thickening. CT also revealed a dilated inferior vena cava (IVC), aneurysmal dilated SVC (7.4-cm diameter, Fig. 3B), and a thrombus in the SVC and left inferior pulmonary artery.

Cardiac catheterisation showed the shape of the CS aneurysm (Fig. 2B). The right heart pressure was recorded, including pulmonary arterial pressure (25/22 mmHg), right ventricular pressure (25/21 mmHg), mean right atrial pressure (23 mmHg) and mean CS aneurysm pressure (22 mmHg). The family declined a request for limited biopsy. No other associated cardiac abnormalities or defects were noted.

Based on the TDI evidence of intact active relaxation and the presence of diastolic equalisation of pressures, it was decided that right heart diastolic failure was the dominant factor contributing to the patient’s symptoms.

**Fig. 4:**
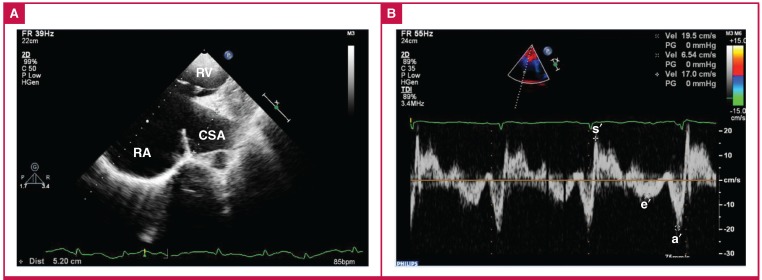
Transthoracic echocardiography performed four years prior to presentation showed: A. the aneurysmal dilated CS was 5 cm in diameter; B. tissue Doppler imaging velocities at the septal mitral annulus: early diastolic myocardial velocity (e′) was 6.54 cm/s and systolic myocardial velocity (s′) was 19.5 cm/s. RV, right ventricle; RA, right atrium; CSA, coronary sinus aneurysm; a′, late diastolic myocardial velocity.

We reviewed the patient’s earlier images. Transthoracic echocardiography performed four years prior to presentation showed that the aneurysmal dilated CS was 5 cm in diameter (Fig. 4B) and the dilated SVC was 3 cm in diameter. Septal mitral annulus e′ and s′ velocity were 6.54 and 19.5 cm/s, respectively (Fig. 4A). These features suggested that the patient had had a long history of right heart diastolic failure, and this may have contributed to the progression of the combined giant aneurysms.

A diagnosis of combined giant aneurysms of the CS and SVC accompanied by right heart failure was made. Due to the two giant venous aneurysms, cardiac thrombosis and severe right heart failure, cardiac transplantation was considered the only viable option. At the time of publication of this report, the patient was being maintained on aspirin and furosemide while awaiting cardiac transplantation.

## Discussion 

On presentation, a physical examination revealed severe facial and lower-extremity oedema, hepatomegaly and ascites. She had no cardiac murmur and no cyanosis, with an oxygen saturation of 96.6% on room air. Venous aneurysms are uncommon, particularly those involving the CS or SVC. The aetiologies of venous aneurysms are still under debate. The causes of acquired venous aneurysms may include trauma, inflammation or pathological processes that affect the vascular wall.4 Longstanding venous hypertension secondary to heart failure, tricuspid valve lesions, cardiomyopathy and constrictive pericarditis have also been considered to cause vascular damage.^5^

Mahmud and colleagues found that CS size was positively correlated with right atrial size and pressure.6 Dilatation of the vena cava may be observed in right heart failure. Wells et al. reported a massive aneurysm of the IVC, and the patient’s longstanding right heart failure was presumed to be the underlying cause.^7^

We report a case of combined giant venous aneurysms, diagnosed using multiple imaging modalities, which provided strong evidence for an aetiology of longstanding right heart diastolic failure.

Aneurysm of the CS is a rare abnormality of the intracardiac vein.^8^ Previous cases in the literature were considered to be congenital or secondary to anomalous drainage.^9^,^10^ We excluded the possibility of congenital causes on the basis of previous CT examinations. Echocardiography and contrast-enhanced CT gave a precise anatomical view of the CS and SVC, demonstrating the absence of anomalous drainage.

Constrictive pericarditis (CP) is pathologically characterised by scarring and a loss of pericardium elasticity, resulting in an external interruption of cardiac filling.^11^ Pericardiectomy remains the most effective therapy for CP. Diastolic dysfunction and low-output syndrome occur in a considerable number of patients after pericardiectomy, which may be the result of atrophic changes in the myocardium associated with longstanding pericardial restriction.^12^ Our patient had a long history of CP before pericardiectomy. On the basis of previous echocardiography, we concluded that the patient had had a long history of right heart diastolic failure.

Echocardiography also revealed the progression of combined venous aneurysms. Colour Doppler echocardiography revealed to-and-fro flow between the CS aneurysm and right atrium. Catheter examination demonstrated equally increased pressure in the right atrium and CS aneurysm. Therefore, it was reasonable to presume that the main cause of the two venous aneurysms was longstanding right heart diastolic failure. Based on the high pressure of the two aneurysms and complications of severe right heart failure, cardiac transplantation may be the only treatment option for this patient.

TDI was helpful in the diagnosis. TDI is a contemporary echocardiographic tool that allows the measurement of intrinsic myocardial velocity. The e′ velocity reflects early diastolic ventricular relaxation in the longitudinal plane, and the s′velocity reflects systolic function in the longitudinal plane. Values of e′ (septal) < 8 cm/s are suggestive of impaired myocardial relaxation.^13^ In this patient, two TDI echocardiography examinations both showed a reduced e′ velocity and a normal s′ velocity. These results strongly suggested a long history of right heart diastolic failure.

## Conclusion

To our knowledge, this is the first reported case of simultaneous presentation of giant aneurysms of the coronary sinus and superior vena cava. Acquired venous aneurysms may result from longstanding right heart failure. The combination of echocardiography, contrast-enhanced CT and cardiac catheterisation facilitated the diagnosis of combined giant venous aneurysms and provided strong evidence for this aetiology.
